# Vitamin D expenditure is not altered in pregnancy and lactation despite changes in vitamin D metabolite concentrations

**DOI:** 10.1038/srep26795

**Published:** 2016-05-25

**Authors:** Kerry S Jones, Shima Assar, Ann Prentice, Inez Schoenmakers

**Affiliations:** 1 MRC Human Nutrition Research, Elsie Widdowson Laboratory, Fulbourn Road, Cambridge, CB1 9NL, UK; 2 MRC Keneba, MRC Unit, Banjul, The Gambia

## Abstract

Pregnancy and lactation are associated with changes in vitamin D and calcium metabolism but the impact of these changes on vitamin D expenditure is unknown. We measured plasma 25(OH)D_3_ half-life with a stable-isotope tracer and investigated relationships with vitamin D metabolites in pregnant, lactating and ‘non-pregnant, non-lactating’ (NPNL) women. Vitamin D metabolites, vitamin D binding protein (DBP), PTH and 25(OH)D_3_ half-life were measured in third-trimester pregnant women (*n*22) and repeated during lactation 12 weeks post-partum (*n*14) and twice in NPNL women (*n*23 and *n*10, respectively) in rural Gambia where calcium intakes are low with little seasonality in UVB-exposure. 25(OH)D_3_ half-life was not significantly different between groups (mean(SD): 20.6(6.8), 22.6(7.7), 18.0(4.7) and 17.7(9.5) days in pregnant, lactating and NPNL women, respectively). Plasma 25(OH)D_3_, 1,25(OH)_2_D, and DBP were higher in pregnancy, and calculated free-25(OH)D_3_ and PTH were lower (*P* < 0.05). In lactation, 25(OH)D_3_ and 24,25(OH)_2_D_3_ were lower compared to pregnant (*P* < 0.001, P = 0.02) and NPNL women (*P* = 0.04, *P* = 0.07). Significant associations were observed between half-life and 25(OH)D_3_ (+ve) in pregnancy, and in all groups between 25(OH)D_3_ and free-25(OH)D_3_ (+ve) and PTH and 25(OH)D_3_ (−ve) (*P* < 0.0001). These data suggest that adaptive changes in pregnancy and lactation occur that prevent pronounced changes in vitamin D expenditure.

Pregnancy and lactation are associated with profound changes in vitamin D, calcium and bone mineral status and metabolism^1^. Knowledge gaps exist around the impact of these changes on vitamin D metabolism and expenditure and whether vitamin D tissue requirements are increased or decreased in pregnancy and lactation^2^.

Vitamin D binding protein (DBP) and 1,25-dihydroxyvitamin D (1,25(OH)_2_D) are elevated from early pregnancy, but the effect of these changes on the circulating concentration of 25(OH)D, the primary vitamin D status marker, is not clear. Recent reviews concluded that there are generally no substantial changes^3^,^4^ and longitudinal studies have shown both increases^5^ and decreases^6^ from early to late pregnancy. Elevation of 1,25(OH)_2_D may be expected to increase 25(OH)D usage directly and potentially result in a decrease in the 25(OH)D body pool^7^,^8^,^9^,^10^ and an increase in 24,25(OH)_2_D production. In addition, the maternal body pool may decrease due to placental metabolism and transfer to the fetus and into breast milk^11^. Stable isotope studies in a rat model suggested that 25(OH)D_3_ half-life was shorter in pregnancy compared to control rats and indicated a specific enhancement of vitamin D transfer to the fetus in the third trimester^12^. Such changes are complex to investigate, however, as they may be affected by many factors. Physiological changes in pregnancy, such as changes in plasma volume, body weight, body composition and renal and hepatic blood flows^13^, may alter vitamin D metabolism and interpretation of vitamin D status markers^14^. In addition, as in a non-pregnant population, changes in 25(OH)D concentration over a period of time are affected by seasonal changes in cutaneous vitamin D production and intake and therefore their separate influences on vitamin D metabolism may be difficult to resolve.

Uncertainties in our understanding of vitamin D metabolism and requirements in pregnancy and lactation are reflected in the range of national and international policies on vitamin D supplementation^2^,^15^,^16^,^17^,^18^. The differing recommendations may reflect different local environments and interpretation of conflicting evidence on the possible negative effect of vitamin D deficiency for maternal, fetal and infant health outcomes. In addition, there are only a few large, well-designed randomized clinical trials (RCT) on the potential benefits or risks of raising vitamin D status in pregnancy^19^,^20^ whilst other RCTs are ongoing (www.clinicaltrials.gov).

Here, we present data of a direct measurement of 25(OH)D expenditure by measuring the plasma half-life of stable isotope labelled 25(OH)D^21^,^22^. We conducted studies in pregnant, lactating and non-pregnant, non-lactating (NPNL) women in a combined longitudinal and cross-sectional design to investigate 25(OH)D expenditure and relationships between vitamin D metabolites, calculated free 25(OH)D and PTH. This study was performed in The Gambia, West Africa, where calcium intakes are low by international standards^23^ and where plentiful tropical UVB sunshine and local dress code allow for relatively constant cutaneous vitamin D synthesis throughout the year, thereby limiting seasonal changes in vitamin D supply and status^24^.

## Results

### Group differences

**Figure 1:**
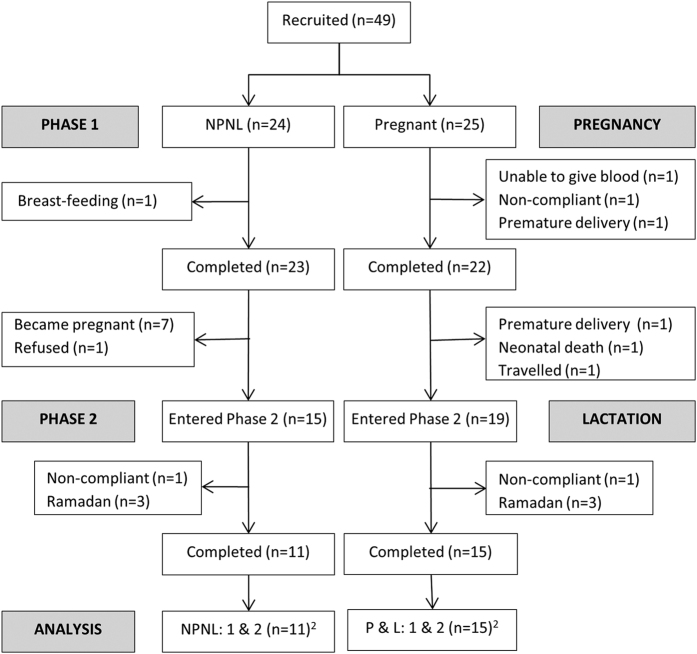
Overview of study design, participant recruitment and follow up^1^. ^1^Numbers (n) refer to data available for the primary outcome of 25(OH)D_3_ half-life. Larger groups sizes are referred to throughout the text for the other measurements and outcomes. ^2^Outlying values were excluded from further analysis in lactation (n = 1) and NPNL2 (n = 1).

**Figure 2:**
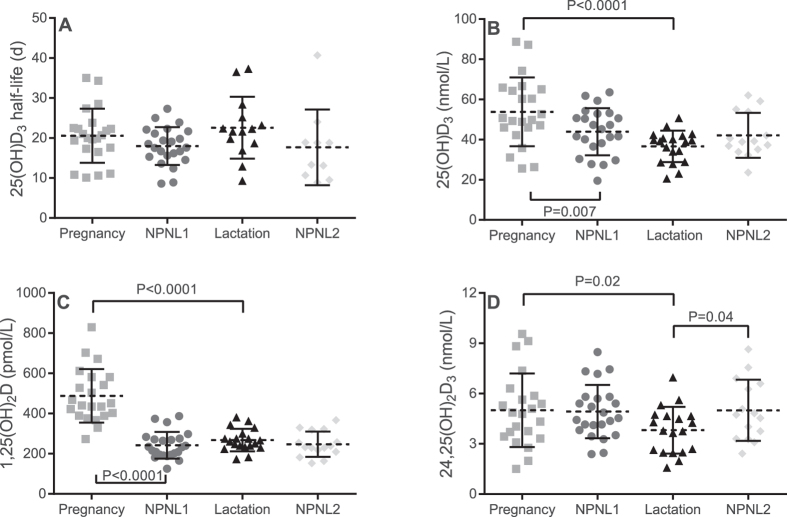
Individual, mean (dashed line) and standard deviation for (**A**) 25(OH)D_3_ half-life, (**B**) 25(OH)D_3_, (**C**) 1,25(OH)_2_D and (**D**) 24,25(OH)_2_D_3_ in pregnant, lactating and non-pregnant non-lactating (NPNL) women.

Forty-five women (*n* = 22 pregnant and *n* = 23 NPNL) completed Phase 1. Nineteen lactating and 15 NPNL women commenced repeat measurements in Phase 2. Of these, 15 and 11, respectively completed the study with a sufficient number of samples (≥4) to calculate 25(OH)D_3_ half-life (Fig. 1). Group means and between-group differences as tested by the linear mixed model are shown in Table 1 and Fig. 2.

Age was not different between groups and, as expected, weight was higher in pregnancy and had decreased by 12 wk post-partum compared to NPNL women. Although 25(OH)D_3_ half-life tended to be longer in pregnancy and lactation compared to NPNL women, the differences were not statistically significant (*P* = 0.2 and *P* = 0.1, respectively). There was also no significant difference in 25(OH)D_3_ half-life within groups between time points (pregnancy to lactation *P* = 0.4; NPNL *P* = 1.0).

Plasma 25(OH)D_3_ was significantly higher in pregnant compared to lactating and NPNL women in the mixed model. Where paired measurements existed between pregnancy and lactation, there was a decline in 25(OH)D_3_ concentration in 18 of 19 women and an average difference between pregnancy and lactation of 18 (±13) nmol/L (P < 0.0001). In contrast, 25(OH)D_3_ concentration remained unchanged in NPNL women between Phase 1 (NPNL1) and 2 (NPNL2) (∆ 0.7 (7.4) nmol/L; *P* = 0.7). Similarly, there was a decrease in 24,25(OH)_2_D_3_ between pregnancy and lactation but not between NPNL1 and 2 (Fig. 2). The ratio of 24,25(OH)_2_D_3_ to 25(OH)D_3_ was not different between pregnancy and NPNL1, lactating women and NPNL2 or between pregnancy and lactation (*P* = 0.2, *P* = 0.3 and *P* = 0.7, respectively).

We observed higher plasma 1,25(OH)_2_D and DBP concentration and lower plasma albumin concentration in pregnancy compared to the other groups. Calculated free- and bioavailable 25(OH)D_3_ were lower in pregnancy and lactation than NPNL (Table 1), reflecting the concurrent doubling of plasma DBP concentration in pregnancy and the observed decrease in 25(OH)D_3_ in lactation, respectively. Plasma PTH concentration was significantly lower in pregnant and higher in lactating women compared to NPNL women. Urinary cAMP/GFR was higher in pregnancy compared to lactating women. Although albumin-corrected plasma Ca was higher in pregnant compared to NPNL women, probably due to much lower plasma albumin, there was no difference in ionized calcium, or plasma phosphate between these two groups. Urinary calcium and phosphate excretion were respectively, higher in lactation compared to pregnancy and in lactating compared to NPNL women. Nutrient intakes were comparable between groups with the exception of higher carbohydrate intake and a trend towards overall higher energy intake in lactation compared to pregnancy.

Results were consistent between the linear model that included all participants and when analysis was restricted only to those who had measures at both time points, i.e. between pregnancy and lactation (Table 2) and between NPNL1 and NPNL2 (Table 3).

### Determinants of 25(OH)D_3_ half-life

Results of the regression analyses of determinants of 25(OH)D_3_ half-life are presented in Table 4. In pregnancy, 25(OH)D_3_ half-life was significantly and positively associated with 25(OH)D_3_ plasma concentration, and there was a trend for the same association with free 25(OH)D_3_ (*P* = 0.08). In NPNL1, there was a trend for a positive association between PTH and half-life (*P* = 0.08). The same relationship was negative and non-significant in pregnancy (β −3.36 (SE 2.75); *P* = 0.2); the NPNL1 and pregnancy slopes were significant different (*P* for interaction, 0.04). There were no other significant relationships.

### Associations between vitamin D metabolites and PTH

**Figure 3:**
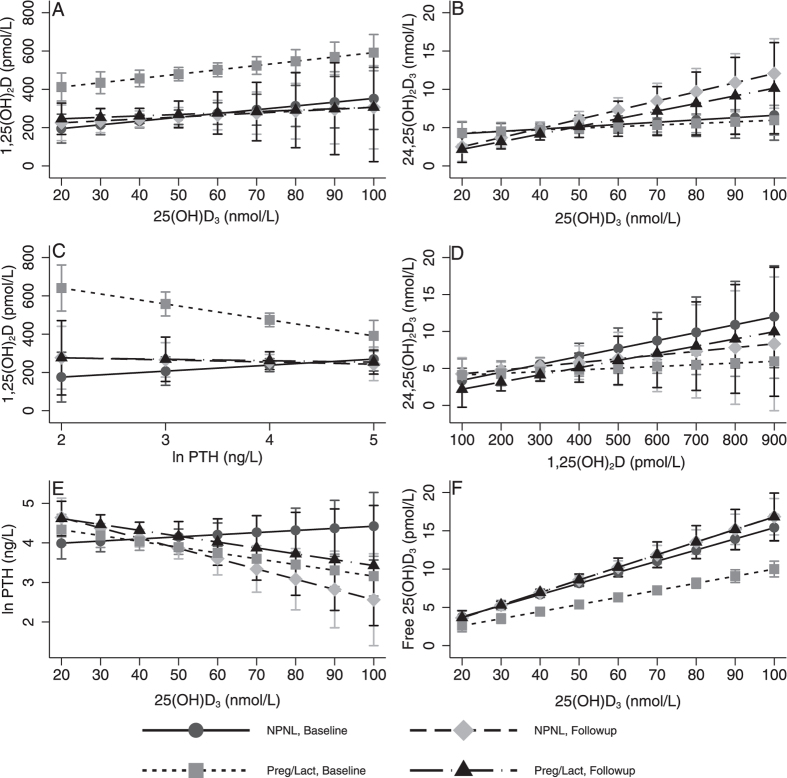
Predicted relationships from mixed linear regression models between vitamin D metabolites and PTH in pregnant, lactating and non-pregnant, non-lactating women (NPNL). Markers with error bars (95% confidence interval) indicate predicted value for *y* variable for given *x* value over the range of observed *x* values in this study. Significant relationships (*P* < 0.05) were seen in pregnancy between 1,25(OH)_2_D and 25(OH)D_3_ (β 2.25 (SE 0.98); *P* = 0.02), 1,25(OH)_2_D and PTH and (β –83 (SE 32); *P* = 0.009) and PTH and 25(OH)D_3_ (β −0.015 (SE 0.005); *P* = 0.005). Also between 24,25(OH)_2_D_3_ and 25(OH)D_3_ for NPNL2 (β 0.12 (SE 0.04); *P* = 0.002) and lactation (β 0.10 (SE 0.05); *P* = 0.04) and between 24,25(OH)_2_D_3_ and 1,25(OH)_2_D for NPNL1 (β 0.011 (SE 0.005); *P* = 0.04). Significant differences between slopes (indicated by significant interaction term) were observed between pregnancy and NPNL1 in the PTH-25(OH)D_3_ relationship (*P* = 0.03), 1,25(OH)_2_D-PTH relationship (*P* = 0.009) and free 25(OH)D_3_-25(OH)D_3_ relationship (*P* = 0.005) and between pregnancy and lactation in the 1,25(OH)_2_D-PTH relationship (*P* = 0.006) and free 25(OH)D_3_-25(OH)D_3_ relationship (*P* = 0.003).

We compared relationships between individual vitamin D metabolites and between PTH and individual vitamin D metabolites in each group (Fig. 3). The positive association between 1,25(OH)_2_D and 25(OH)D_3_ observed in all groups was only significant in pregnancy (β 2.25 (SE 0.98); *P* = 0.02) (Fig. 3A). The positive associations between 24,25(OH)_2_D_3_ and 25(OH)D_3_ were significant in lactation (β 0.10 (SE 0.05); *P* = 0.04) and in NPNL2 (β 0.12 (SE 0.04); *P* = 0.002) (Fig. 3B). Similarly, there were positive relationships between 24,25(OH)_2_D_3_ and 1,25(OH)_2_D (Fig. 3D) which were significant for NPNL1 only (β 0.01 (SE 0.01); *P* = 0.04). In pregnancy, 1,25(OH)_2_D concentration was strongly negatively associated with PTH (β −83 (SE 32); *P* = 0.009) and this relationship was significantly different from that for NPNL1 (*P* = 0.009) (Fig. 3C). PTH was negatively associated with 25(OH)D_3_ in pregnancy (β −0.015 (SE 0.005); *P* = 0.005) and for NPNL2 women (β −0.026 (SE 0.010); *P* = 0.01) and these relationships were significantly different (*P* < 0.05) from the non-significant slope observed for NPNL1 women (β 0.005 (SE 0.008); *P* = 0.5). There was a strongly significant (*P* < 0.0001) positive relationship between total and free 25(OH)D_3_ in all groups. In pregnancy, for a given concentration of 25(OH)D_3_, free 25(OHD_3_ was significantly lower than in other groups as indicated by significant differences between the slopes (*P* < 0.0001) (Fig. 3F). Very similar relationships were observed if either 1,25(OH)_2_D or PTH were included in the models as covariates or when free 25(OH)D_3_ instead of 25(OH)D_3_ was used in the same regression models (data not shown).

## Discussion

We found no significant differences in 25(OH)D_3_ half-life between pregnant, lactating and NPNL women. There were differences in the relationships between 25(OH)D_3_ and 1,25(OH)D_3_ with PTH in pregnancy compared to the other groups. Free 25(OH)D_3_ was lower in pregnancy suggesting that availability of 25(OH)D may be lower. These pregnancy-induced changes did not appear to markedly affect 25(OH)_3_ expenditure. Adaptation of vitamin D metabolism in response to pregnancy and lactation may prevent pronounced changes in vitamin D expenditure. In the following paragraphs, we discuss the potential physiological mechanisms and relevance of this apparent paradoxical finding.

Despite the characteristic^3^,^25^ increase in plasma 1,25(OH)_2_D in pregnancy, we observed no significant differences in 25(OH)D_3_ half-life between groups and, although not significant, the results suggest that 25(OH)D_3_ half-life may be longer in pregnancy and lactation. The factors that cause increased circulating 1,25(OH)_2_D in pregnancy are unclear^26^. Although PTH levels are generally lower in pregnancy, parathyroid hormone related peptide (PTHrP), its close homologue, is elevated, activates the PTH/PTHrP receptor and stimulates renal 1,25(OH)_2_D production, albeit less strongly than PTH^11^,^27^. Consistent with this, urinary cAMP/GFR was higher in pregnancy, indicative of an upregulation of PTH/PTHrP-dependent activity. Increases in oestradiol, placental lactogen or prolactin may also contribute to the increase in 1,25(OH)_2_D concentration^28^,^29^. However, even in pregnancy, circulating 1,25(OH)_2_D is around 100-fold lower than 25(OH)D, and it is possible that much larger changes in 1,25(OH)_2_D would be necessary for an observable change in 25(OH)D_3_ half-life.

Suppression of renal CYP24A1 activity in pregnancy may play a role in determining vitamin D expenditure. PTH downregulates renal CYP24A1 activity^30^ and animal studies suggest that PTHrP may have a similar effect^26^. Typically, higher 24,25(OH)_2_D is associated with higher 25(OH)D and 1,25(OH)_2_D concentration, the latter of which upregulates CYP24A1 activity^21^,^31^. In support of a suppressive effect of PTHrP on CYP24A1, we found no difference in 24,25(OH)_2_D_3_ concentration in pregnancy compared to NPNL1 despite higher 25(OH)D_3_ and 1,25(OH)_2_D. The 24-hydroxylation pathway may be quantitatively more relevant to 25(OH)D metabolism than the 1,25(OH)_2_D pathway, thus PTHrP suppression of CYP24A1 may provide further explanation for our 25(OH)D_3_ half-life results. Because of the suppression of CYP24A1 in pregnancy plasma 1,25(OH)_2_D may, as observed in Fig. 3, more closely reflect PTH/PTHrP activity than in the non-pregnant state.

As in other pregnancy studies, we observed that DBP plasma concentration was higher in pregnant compared to lactating and NPNL women. Plasma 1,25(OH)_2_D concentration is tightly regulated and the concomitant rise in 1,25(OH)_2_D may occur to maintain free 1,25(OH)_2_D concentration^3^,^29^. An equivalent rise in 25(OH)D concentration is probably not generally observed because it is not under strict homeostatic control and largely depends on vitamin D supply. The increase in DBP concentration may provide a counterbalance to drivers of increased utilization of 25(OH)D and act as a mechanism to conserve 25(OH)D during pregnancy and prolong 25(OH)D_3_ half-life despite increased 1,25(OH)_2_D. We have shown previously a significant positive relationship between DBP concentration and 25(OH)D_3_ half-life^22^, thus the higher DBP concentration in pregnancy may reduce availability of 25(OH)D. Although the association with DBP was not significant in this study, the direction and magnitude of the relationship was as reported previously^22^.

In common with all studies of pregnancy, the interpretation of biochemical parameters is complex due to the effects of hemodilution, changes in renal and hepatic clearance and the influence of pregnancy specific hormones^32^ together with genetic and epigenetic variation in genes controlling their production and activity^33^. Our data show no change in vitamin D status in NPNL women between phases and is consistent with the assumed constant vitamin D supply in The Gambia. This therefore suggests that the changes observed in 25(OH)D_3_ concentration in pregnancy represent a genuine increase in 25(OH)D_3_ concentration.

In lactation, 25(OH)D_3_ concentration was lower compared to the other groups and suggests a decrease in vitamin D status in lactation. Similar findings were presented in a study of lactating Tanzanian women with higher plasma 25(OH) than women in this study^34^. Other studies have suggested that 25(OH)D concentration may be decreased only after extended breast-feeding^35^,^36^. As cholecalciferol is the major vitamin D metabolite in breast milk^2^, this may explain the paradox of lower 25(OH)D_3_ concentration but no significant difference in 25(OH)D_3_ half-life in lactation. Transfer of cholecalciferol into breast milk and its effect on endogenous 25(OH)D would not be discernible in 25(OH)D_3_ half-life because we gave 25(OH)D_3_ rather than its precursor. In contrast to studies in Western populations (reviewed in^37^) but consistent with studies in Gambian women and likely related to low calcium intakes^25^, we found elevated PTH in lactation. Despite this, 1,25(OH)_2_D was not significantly different in lactation and, together with DBP and albumin, had decreased from pregnancy to concentrations comparable to NPNL women. In lactation, 24,25(OH)_2_D_3_ concentration was lower compared to the other groups but the 24,25(OH)_2_D_3_:25(OH)D_3_ ratio was not different. This suggests that beyond the precursor-product relationship between these two metabolites^31^ there is no independent effect of lactation on vitamin D catabolism.

Free 25(OH)D and 1,25(OH)_2_D may provide alternative markers of vitamin D availability and biological activity^38^ and may be important determinants of 25(OH)D metabolism. In general, although calculated and directly measured free 25(OH)D correlate well^39^,^40^ this may not be true in all physiological conditions, particularly in situations where DBP is substantially elevated or decreased since the calculation of free 25(OH)D requires DBP, albumin and 25(OH)D concentrations^39^. In pregnancy and lactation, data on the changes in free 25(OH)D and free 1,25(OH)_2_D levels are limited, particularly their direct measurement. In our study, calculated free 25(OH)D_3_ was lower in pregnancy (due to much higher DBP concentration) but direct measurement of free 25(OH)D demonstrated no difference between pregnant and non-pregnant US white women^39^,^41^. This is in contrast to measured free 1,25(OH)_2_D that was higher in pregnancy^42^. Whether changes in circulating albumin or lipoproteins, or changes in the affinity of DBP for vitamin D metabolites in pregnancy^42^ affect levels of free vitamin D metabolites is unknown and is an area for future study, particularly with respect to placental metabolism, fetal transfer and vitamin D status at birth.

Nutrient intakes including energy, calcium and phosphorus were similar between groups although there was a trend for higher energy intake during lactation. Energy intakes in pregnancy and lactation and gestational weight gain in this study were below international recommendations and observed Western norms^43^. The women in this study were from a well-characterised marginally nourished population and seasonal fluctuations in energy balance^44^ mean that some women may have been in negative energy balance. However, the observed maternal weight gains are consistent with other cohorts from this population^45^,^46^ and energy-sparing alterations in maternal metabolism in under-nourished populations are thought to negate some of the additional energy costs associated with pregnancy^45^. Because of the possible negative energy balance it is not appropriate to adjust for underreporting. Calcium intakes, although low by Western standards, were typical for this population^47^. Sources of calcium in rural Gambia are green vegetables, indigenous leaves and plants and small fish. These foods are not significant sources of energy or macronutrients (e.g. dairy products) and therefore calcium intakes are unlikely to be affected by any underreporting of total energy intake.

Study strengths were the minimal seasonal influence on vitamin D status and a relatively modest influence of other factors because this population is characterized by a high frequency of GC-1F1F and 1F1S genotypes^22^,^48^ with a more uniform calcium intake and dietary pattern compared to Western populations. We maximized statistical power, and further minimized the influence of season, by studying the same women through pregnancy and lactation and, at the same time, matched NPNL women. The sample size was based on our previous studies and the power to detect a 2.5 d difference in 25(OH)D_3_ half-life. Despite observing a difference of this magnitude, the result was not significant and may be partly explained by a larger within-population variation^22^. Using the within group SD of 5.75 d observed here, we estimate that we would be able to detect a group difference of 5 d (α of 0.05 and 80% power) with a sample size of 22 participants per group. To observe a difference of 2.5 d, we would need 84 participants per group. A limitation of the study was the higher than expected attrition between phases and the consequently smaller number of participants followed through to lactation and second measurements in NPNL women particularly for measurement of 25(OH)D_3_ half-life. Free 25(OH)D concentration was calculated and not measured directly and there may be discrepancies between these values during pregnancy^39^. We did not quantify plasma 25(OH)D_2_ but our previous data suggest the contribution to total 25(OH)D is very small in this population^22^,^49^.

In conclusion, in women with low dietary calcium intakes, year-round availability of UVB and stable vitamin D status, 25(OH)D_3_ half-life was not changed in pregnancy or lactation compared to non-pregnant women. There were differences in vitamin D metabolite, DBP and PTH concentrations between groups and, in pregnancy and lactation, in the relationships between PTH and vitamin D metabolites, and in pregnancy between free 25(OH)D_3_ and 25(OH)D_3_. These data suggest that adaptive changes in pregnancy and lactation occur that prevent pronounced changes in vitamin D expenditure and depletion of vitamin D stores. These findings need replication in populations with different genetic background, particularly in relation to DBP, or in vitamin D deficiency.

## Methods

### Study setting

The study was performed between November 2011 and October 2012 at MRC Keneba, a UK Medical Research Council field station in the West Kiang region of The Gambia (latitude 13°N), primarily a rural subsistence farming community^50^. The study was conducted according to the guidelines laid down in the Declaration of Helsinki and all procedures involving the participants were conducted as approved by the joint Gambian Government-MRC Ethics Committee. Trained staff explained the study to the participants and informed, written consent was obtained.

### Participants and recruitment

The study design was to measure 25(OH)D_3_ half-life twice in each woman, once during late gestation (Phase 1) and once in lactation (Phase 2). A control group of NPNL women was also measured twice, once at each phase. The number of participants was selected based on data from our previous studies of 25(OH)D_2_ half-life in men from the same population^49^. We estimated a sample size of 18 women per group was needed to detect a mean difference in 25(OH)D_3_ half-life between groups (e.g. pregnant and NPNL women) of 20% using an α of 0.05 and 80% power and based on a standard deviation of 2.7 days. Additional women were recruited at Phase 1 to allow for attrition between Phases 1 and 2. Participants (*n* = 45) were healthy females, aged between 18 and 47 years at their first measurement. Pregnant women were identified through the screening and recruitment protocols of the ENID trial^51^. Women ineligible for the ENID trial due to presentation later than 20 weeks (wk) gestational age (GA) (assessed by ultra-sound) were subsequently assessed for eligibility to this study. Inclusion criteria for pregnant women were presentation with a singleton fetus between 20 and 30 wk GA and, for follow-up measurements in lactation, the infant must have been exclusively breast-fed up to (three months) and during the study period (one month), which is typical in this setting. Following recruitment of a pregnant woman, an age- and parity-matched NPNL woman was identified from The West Kiang Demographic Surveillance System^50^ and invited to participate. NPNL was defined as not currently pregnant nor having breast fed in the previous three months as assessed by self-reporting during interview with field staff. Exclusion criteria for all participants were severe anaemia (haemoglobin <7 g/dL), known sickle cell disease, known history of liver, kidney, gut or malabsorption problems or any other chronic condition, on prescription medicine, regular consumption of vitamin D supplements, a broken bone in the past three years, recent illness or infection, reported to be past menopause or recent malaria parasitemia. In addition, pregnant women were excluded for any other pregnancy-related complication or other reason on the discretion of their midwife.

### Study protocols

Phase 1 measurements were conducted between November 2011 and July 2012 and Phase 2 measurements between April and October 2012. In Phase 1, the study protocol commenced at 30 wk GA. For women who also agreed to take part in Phase 2, measurements were started 12 wk post-partum. In each phase, matched NPNL women were studied at the same time to control for possible influences of season on vitamin D metabolites as well as seasonal changes in food intake, body composition and other factors. Procedures on day 1 and day 21 were performed at the MRC Keneba field station. On day 1, after an overnight fast and voiding of the first morning urine, a timed 2-hour fasting urine was collected from approximately 7.00 a.m. with a antecubital venous blood sample collected after 1 hour into tubes containing lithium heparin (LH) or ethylenediaminetetraacetic acid (EDTA) anticoagulant (S-Monovettes, Sarstedt Ltd, Leicester, UK). Height (Leicester Stadiometer, Chasmoors Ltd, UK), weight (Tanita HD305 scale, Tanita, Amsterdam, The Netherlands) and mid-upper arm circumference (MUAC) were measured using standardized protocols. After completion of the urine collection, the participant was given an oral tracer dose of 40 nmol (16 μg) of stable isotope labelled 25(OH)D_3_ (d_3_-25(OH)D_3_) (deuterated (6, 19, 19) 25-hydroxy vitamin D_3_ (product number: 705888; 97 atom %; purity 98%; Sigma-Aldrich, Poole, UK) dissolved in vegetable oil with a standard breakfast^22^. Further fasted blood samples were collected in the participant’s home into LH tubes on day 6 and days 9, 21, 24, 27 and 30 (+/−2 days) for measurement of the plasma tracer concentration for the calculation 25(OH)D_3_ half-life. The 2-hour fasting urine with timed blood sample protocol was repeated on the day 21 visit. During the 30 day study period, dietary nutrient intakes were quantitatively assessed by 2-day weighed food diary^21^ and nutrient intakes calculated using the DINO (Diet In Nutrients Out) analysis programme^52^,^53^.

### Sample processing and laboratory analysis

Ionized calcium was measured in freshly collected EDTA whole blood on days 1 and 21; values corrected to pH 7.4 are reported (Radiometer UK Ltd, Crawley, UK). Plasma was separated by centrifugation at 4 °C within one hour of sample collection and frozen at −70 °C. Collected urine was mixed thoroughly, acidified (concentrated hydrochloric acid, 10ml/l Fisher Scientific) and aliquots stored at −20 °C. Samples were transported to MRC Human Nutrition Research frozen on dry ice and subsequently stored at −80 °C (plasma) or −20 °C (urine) until analysis.

Plasma 25(OH)D_3_, 24,25(OH)D_3_ and d_3_-25(OH)D_3_ were measured by UPLC-MS/MS as described previously^22^ with slight modifications to the mass spectrometer parameters to include 24,25(OH)D_3_ quantified using d_6_-24,25(OH)D_3_ as internal standard and the mass transitions of 623.1 > 298 and 629.2 > 298 m/z, respectively. Intra- and inter-assay coefficients of variation were <10% for all analytes. Measured concentrations of 25(OH)D_3_ in NIST standards (National Institute of Standards and Technology, Gaithersburg, MD, USA) were within 10% of the reference value. Plasma calcium, phosphate, creatinine and albumin and urinary calcium (*u*Ca), phosphate (*u*P) and creatinine (*u*Cr) were measured on the Kone Lab 20i clinical chemistry analyser platform (Kone, Espoo, Finland) where intra-assay and inter-assay % coefficient of variation (CV) was ≤2% and <5%, respectively. Plasma 1,25(OH)_2_D was measured with a radioimmunoassay (IDS Ltd., Tyne and Wear, UK) and DBP by an ELISA with polyclonal antibodies^40^ (Immundiagnostik AG, Bensheim, Germany). These assays both had intra- and inter-assay %CVs of ≤3% and <9%. Urinary cyclic adenosine monophosphate (cAMP) was measured by ELISA (GE Healthcare Life Sciences, Buckinghamshire, UK) and had an intra-assay CV of 7% and inter-assay CV of 19%. All analyses were performed in duplicate with LH plasma with the exception of PTH that was measured in singleton in EDTA plasma by immunoassay (Immulite; Siemens Healthcare Diagnostics Ltd) and had a between-assay CV of <4%. Assay performance was monitored using kit and in-house controls and under strict standardization according to ISO 9001:2000. Quality assurance of 25(OH)D_3_, 1,25(OH)_2_D and PTH assays was performed as part of the Vitamin D External Quality Assessment Scheme (www.deqas.org) and the National External Quality Assessment Scheme (www.ukneqas.org.uk), and were within accepted limits. In addition, an aliquot of a pooled plasma sample was assayed in each batch to monitor possible drift over time and to provide running quality assurance for analytes where no external reference material was available.

### Data analysis

25(OH)D_3_ half-life was calculated from the slope of plasma d_3_-25(OH)D_3_ disappearance using the line of best fit of the natural log of d_3_-25(OH)D_3_ concentration against time^22^. The ratio of 24,25(OH)_2_D_3_ to 25(OH)D_3_ was calculated as a marker of vitamin D catabolism^54^. Plasma calcium was adjusted for albumin (Ca_alb_) as described previously^25^. Fasting 2-h *u*Ca and *u*P were corrected for urinary volume by dividing by *u*Cr to derive *u*Ca/*u*Cr and *u*P/*u*Cr ratios. Urinary cAMP was corrected for glomerular filtration rate (GFR) by multiplying urinary cAMP by the quotient of plasma and urinary creatinine^55^,^56^; this measure is considered analogous to nephrogenous cAMP^55^. Free- and bioavailable 25(OH)D_3_ were calculated using the plasma concentrations of 25(OH)D_3_, DBP and albumin^22^. Statistics were performed in Stata 13.1 (StataCorp, TX, USA). There were no differences between day 1 and day 21 values of calcium and vitamin D metabolism markers with paired Student’s t tests, so for each analyte the mean of day 1 and 21 values was calculated and used for subsequent analysis. Data were checked for outliers and normality using box-whisker plots. Normally distributed data are presented as mean and standard deviation (SD). Skewed data were log_e_-transformed and are presented as the geometric mean and 95% confidence interval. Values more than 3*IQR beyond the upper IQR were not included in the analyses (these were two values for 25(OH)D_3_ half-life and one for urinary cAMP).

To maximise statistical power, group differences (pregnant vs lactation, NPNL1 vs NPNL2 and pregnant/lactation vs NPNL1/2) were primarily explored using the whole dataset that included both matched and unmatched participants (due to attrition between phases 1 and 2) (Table 1). We used a linear mixed model consisting of a response variable and fixed effects defined as group (pregnant/lactating or NPNL) and time (Phase 1 or Phase 2). To allow for the influence of repeated measurements within the same individual, participant identity (ID) was included as a random effect. Further pairwise comparison of group means was tested using Stata’s post-regression *margins* command. In addition and to see if our observations were influenced by those individuals who did not take part in both phases, the group differences were also tested between individuals who had paired measures at the two time points with paired Student’s t-tests (Tables 2 and 3).

Predictors of 25(OH)D_3_ half-life and vitamin D metabolite concentrations were investigated using a similar linear mixed model as used for the group comparisons and focused on vitamin D metabolites and PTH. As above, fixed effects were group and phase, and participant ID was included as a random effect. Interaction terms were included between the continuous predictor variable and the factorial group and phase variables to test for differences between groups in the relationships between predictor variables and the dependent variable. Slopes and *P* values for each group were generated using the *margins* command and interactions tested using *margins* with *pwcompare* option^57^. For unconverted, normally distributed data the regression coefficient (β) represents the predicted change in *y*-variable for a one unit change in the predictor *x*-variable. For a log_e_ converted *y*-variable, a one unit change in the *x*-variable predicts a percentage change in *y* of the coefficient multiplied by 100%. If the x-variable is logged, then a 1% change in *x* approximately predicts a change in *y* of the coefficient divided by 100. Throughout, a significance level of *P* < 0.05 was used.

## Additional Information

**How to cite this article**: Jones, K. S. *et al*. Vitamin D expenditure is not altered in pregnancy and lactation despite changes in vitamin D metabolite concentrations. *Sci. Rep.* **6**, 26795; doi: 10.1038/srep26795 (2016).

## Figures and Tables

**Table 1:** Participant characteristics, biochemistry and dietary intakes^1^,^2^.

	Pregnancy (*n* = 22)^3^	NPNL1 (*n* = 23)	Lactation (*n* = 19)	NPNL2 (*n* = 15)
Age and anthropometry				
Age at baseline, years	28.6 (7.8)	28.9 (8.0)	27.7 (6.4)	29.3 (8.6)
Haemoglobin, mg/dl	12.3 (1.4)	13.8 (1.8)	Not measured	Not measured
Height, m	1.62 (0.06)^a^	1.61 (0.05)	1.62 (0.06)^a^	1.62 (0.04)
Weight, kg	61.0 (8.0)^a,b^	54.6 (8.6)^a^	57.2 (7.9)^b^	56.1 (10.7)
MUAC, mm	26.9 (2.7)^a^	26.5 (2.9)	27.4 (2.9)^a^	26.5 (3.2)
Plasma analytes				
25(OH)D_3_ half-life, d	20.6 (6.8)	18.0 (4.7)	22.6 (7.7)[14]	17.7 (9.5)[10]
25(OH)D_3_, nmol/L	53.8 (17.1)^a,b^	43.9 (11.7)^a^	36.6 (7.8)^b,^*	42.1 (11.1)*
25(OH)D_3_, nmol/L, *n* (%)				
<30	2 (9)	4 (17)	5 (26)	1 (7)
<50	11 (50)	14 (61)	18 (95)	10 (67)
<75	20 (91)	23 (100)	19 (100)	14 (93)
Free 25(OH)D_3_, pmol/L	5.7 (1.8)^a,b^	7.2 (1.9)^a^	6.3 (1.5)^b,*^	7.3 (2.1)*
Bioavailable 25(OH)D_3_, nmol/L	1.3 (0.4)^a,b^	2.3 (0.6)^a^	2.0 (0.4)^b,c^	2.3 (0.6)^c^
24,25(OH)_2_D_3_, nmol/L	5.0 (2.2)^a^	4.9 (1.6)	3.8 (1.4)^a,b^	5.0 (1.8)^b^
1,25(OH)_2_D_3_, pmol/L	487 (133)^a,b^	241 (67)^a^	268 (56)^b^	247 (63)
Ratio 24,25(OH)_2_/25(OH)D_3_	0.10 (0.06)	0.12 (0.04)	0.11 (0.03)	0.12 (0.03)
PTH, ng/L ^#^	46.4 (37.2, 57.8)^a,^*	61.6 (49.4, 76.9)*	78.4 (64.2, 95.8)^a,b^	56.2 (43.2, 73.1)^b^
DBP, mg/L	775 (128)^a,b^	482 (60)^a^	461 (63)^b^	463 (74)
Albumin, g/L	25.6 (1.94)^a,b^	35.2 (2.71)^a^	35.0 (2.55)^b^	35.0 (2.02)
Ca_alb_, mmol/L	2.42 (0.06)^a,b^	2.32 (0.07)^a,c^	2.34 (0.05)^b^	2.34 (0.05)^c^
Ionised calcium, mmol/L	1.11 (0.03)[21]*	1.11 (0.03)^a^	1.12 (0.03)[16]^*^	1.13 (0.03) [13]^a^
Plasma phosphate, mmol/L	1.05 (0.14)^a^	1.04 (0.09)	1.24 (0.14)^a,b^	1.04 (0.12)^b^
Urinary markers				
Urinary cAMP, nmol/dL GFR^4^	40.3 (9.7) [21]^a^	36.6 (8.6)^b^	31.5 (6.0)^a^	30.9 (8.4)^b^
*u*Ca/*u*Cr, mmol/mmol^#^	0.05 (0.04, 0.07)^a^	0.07 (0.04, 0.10)	0.08 (0.06, 0.13)^a^	0.07 (0.04, 0.10)
*u*P/*u*Cr, mmol/mmol	1.13 (0.40)	1.03 (0.49)	1.29 (0.46)^a^	0.91 (0.41)^a^
Dietary intakes				
Energy, kcals	1535 (440)*	1608 (429)	1743 (471) [18]*	1587 (261) [14]
Protein, g/d	46 (17)	47 (13)	48 (13) [18]	45 (11) [14]
Fat, g/d	39 (20)	34 (23)	32 (17) [18]	35 (13) [14]
Carbohydrate, g/d	278 (69)^a^	287 (67)	329 (91) [18]^a^	296 (52) [14]
Calcium, mg/d	341 (135)*	282 (105)*	305 (119) [18]	263 (85) [14]
Phosphate, mg/d	684 (206)	666 (186)	737 (207) [18]	625 (139) [14]
Iron, mg/d	29 (16)	28 (23)	33 (25) [18]	22 (8) [14]
Zinc, mg/d	7.3 (2.7)	7.2 (2.2	7.7 (2.3) [18]	7.3 (2.0) [14]
Magnesium, mg/d	425 (124)	433 (169)	462 (155) [18]	393 (85) [14]
Potassium, mg/d	1965 (550)	1971 (757)	2105 (643) [18]	1834 (438) [14]

^*^Indicates a trend for a difference between groups (*P* ≥ 0.05 <0.1).

^1^Like subscripts across rows indicates a significant difference (*P* < 0.05). Group comparisons were made with a linear mixed model to allow for matched and unmatched participants using Stata 13.1 (StataCorp). Each model consisted of the response variable, fixed effects of group (pregnancy/lactation or NPNL) and time (Phase 1 or 2). Participant ID was included as a random effect. Post-regression pairwise comparisons were performed between NPNL1 and NPNL2, pregnancy and lactation, NPNL1 and pregnancy, and NPNL2 and lactation.

^2^Data are presented as mean (SD) except variables ^#^natural logarithm adjusted with data presented as geometric mean and 95% confidence interval.

^3^Group sizes (n) are as stated in top row or where different in square brackets against the specific variable.

^4^Urinary cAMP as a function of GFR.

**Table 2:** Participant characteristics, biochemistry and dietary intakes for women with data from pregnancy and lactation^1^^,^^2^.

	Pregnancy (*n* = 19)^3^	Lactation (*n* = 19)	*P*
Age and anthropometry			
Height, m	1.63 (0.06)	1.62 (0.06)	0.011
Weight, kg	60.8 (8.4)	57.2 (7.9)	<0.0001
MUAC, mm	26.7 (2.8)	27.4 (2.9)	0.024
Plasma analytes			
25(OH)D_3_ half-life, d	21.1 (5.5) [14]	22.6 (7.7) [14]	0.54
25(OH)D_3_, nmol/L	54.6 (17.4)	36.6 (7.8)	<0.0001
25(OH)D_3_, nmol/L, *n* (%)			
<30	2 (11%)	5 (26%)	–
<50	9 (47%)	18 (95%)	–
<75	17 (89%)	19 (100%)	–
Free 25(OH)D_3_, pmol/L	5.62 (1.63)	6.33 (1.53)	0.067
Bioavailable 25(OH)D_3_, nmol/L	1.30 (1.12)	2.00 (1.78)	<0.0001
24,25(OH)_2_D_3_, nmol/L	4.93 (2.26)	3.81 (1.40)	0.060
1,25(OH)_2_D_3_, pmol/L	509 (129)	268 (56)	<0.0001
Ratio 24,25(OH)_2_D_3_:25(OH)D_3_	0.10 (0.05)	0.10 (0.03)	0.49
PTH, ng/L^#^	45.2 (35.2, 58.2)	78.4 (64.2)	0.0001
DBP, mg/L	795 (121)	461 (63)	<0.0001
Albumin, g/L	25.5 (1.6)	35.0 (2.5)	<0.0001
Ionised calcium, mmol/L	1.10 (0.03) [15]	1.12 (0.03) [15]	0.09
Plasma phosphate, mmol/L	1.04 (0.13)	1.24 (0.13)	<0.0001
Urinary markers			
Urinary cAMP, nmol/dL GFR^4^	42.4 (13.5)	31.5 (6.0)	0.0016
*u*Ca/*u*Cr, mmol/mmol^#^	0.05 (0.04, 0.07)	0.08 (0.06, 0.13)	0.008
*u*P/*u*Cr, mmol/mmol	1.12 (0.39)	1.29 (0.46)	0.14
Dietary intakes			
Energy, kcals	1530 (412)	1743 (471)	0.17
Protein, g/d	47 (18)	48 (13)	0.80
Fat, g/d	33 (20)	32 (17)	0.90
Carbohydrate, g/d	280 (70)	329 (91)	0.07
Calcium, mg/d	356 (131)	305 (119)	0.28
Phosphate, mg/d	686 (206)	737 (207)	0.43
Iron, mg/d	30 (16)	33 (25)	0.58
Zinc, mg/d	7.4 (2.7)	7.7 (2.3)	0.69
Magnesium, mg/d	425 (129)	462 (155)	0.35
Potassium, mg/d	1950 (560)	2105 (643)	0.35

^1^Groups comparisons performed with Student’s t-test.

^2^Data are presented as mean (SD) except variables ^#^natural logarithm adjusted with data presented as geometric mean and 95% confidence interval.

^3^Groups sizes (n) are as stated in the top row, otherwise in square brackets against each variable.

^4^Urinary cAMP as a function of GFR.

**Table 3:** Participant characteristics, biochemistry and dietary intakes for non-pregnant, non-lactating (NPNL) women with data from two time points^1^^,^^2^.

	NPNL1 (*n* = 14)^3^	NPNL2 (*n* = 14)	*P*
Age and anthropometry			
Height, m	1.62 (0.04)	1.62 (0.04)	0.091
Weight, kg	55.9 (10.2)	56.8 (10.8)	0.07
MUAC, mm	26.8 (3.1)	26.7 (3.2)	0.42
Plasma analytes			
25(OH)D_3_ half-life, d	15.5 (5.1) [10]	17.7 (9.5) [10]	0.52
25(OH)D_3_, nmol/L	41.4 (12.7)	42.1 (11.1)	0.72
25(OH)D_3_, nmol/L, *n* (%)			
<30	4 (29%)	1 (7%)	–
<50	9 (64%)	10 (71%)	–
<75	14 (100%)	14 (100%)	–
Free 25(OH)D_3_, pmol/L	7.0 (2.0)	7.3 (2.1)	0.37
Bioavailable 25(OH)D_3_, nmol/L	2.2 (0.6)	2.3 (0.6)	0.62
24,25(OH)_2_D_3_, nmol/L	4.7 (1.9)	5.1 (1.8)	0.49
1,25(OH)_2_D_3_, pmol/L	219 (62)	239 (56)	0.17
Ratio 24,25(OH)_2_D_3_:25(OH)D_3_	0.12 (0.05)	0.12 (0.03)	0.90
PTH, ng/L^#^	59.9 (42.5, 84.6)	57.9 (43.9, 76.3)	0.84
DBP, mg/L	467 (65)	461 (76)	0.77
Albumin, g/L	35.7 (3.0)	35.0 (2.1)	0.15
Ionised calcium, mmol/L	1.11 (0.03) [12]	1.13 (0.03) [12]	0.051
Plasma phosphate, mmol/L	1.02 (0.08)	1.05 (0.13)	0.49
Urinary markers			
Urinary cAMP, nmol/dL GFR^4^	36.2 (8.0)	30.7 (8.6)	0.11
*u*Ca/*u*Cr, mmol/mmol^#^	0.08 (0.05, 0.13)	0.07 (0.04, 0.11)	0.48
*u*P/*u*Cr, mmol/mmol	1.09 (0.55)	0.87 (0.39)	0.058
Dietary intakes [14 per group]			
Energy, kcals	1589 (437)	1587 (261)	0.98
Protein, g/d	45 (12)	45 (11)	0.90
Fat, g/d	38 (19)	35 (13)	0.52
Carbohydrate, g/d	286 (73)	297 (52)	0.51
Calcium, mg/d	263 (103)	263 (85)	0.99
Phosphate, mg/d	648 (213)	625 (139)	0.70
Iron, mg/d	27 (27)	22 (8)	0.51
Zinc, mg/d	7.0 (2.5)	7.3 (2.0)	0.71
Magnesium, mg/d	418 (168)	393 (85)	0.61
Potassium, mg/d	1879 (700)	1834 (438)	0.84

^1^Groups comparisons performed with Student’s t-test for women at two time points approximately 3 months apart.

^2^Groups sizes (n) are as stated in the top row, otherwise in square brackets against each variable.

^3^Data are presented as mean (SD) except variables ^#^natural logarithm adjusted with data presented as geometric mean and 95% confidence interval.

^4^Urinary cAMP as a function of GFR.

**Table 4:** Linear regression analysis of variables predicting 25(OH)D_3_ half-life^1^.

	Pregnancy (*n* = 22)		NPNL1 (*n* = 23)		Lactation (*n* = 14)		NPNL2 (*n* = 10)	
	β (se)	***P***	β (se)	***P***	β (se)	***P***	β (se)	***P***
25(OH)D_3_, nmol/L	**0.220 (0.080)**	**0.006**	0.009 (0.114)	0.8	−0.031 (0.235)	0.9	−0.045 (0.174)	0.8
Free 25(OH)D_3_, pmol/L	1.372 (0.782)	0.08	−0.301 (0.718)	0.7	−0.185 (1.030)	0.9	−0.811 (0.941)	0.4
24,25(OH)_2_D_3_, nmol/L	0.272 (0.646)	0.7	0.349 (0.870)	0.7	−0.648 (1.348)	0.6	−1.694 (1.260)	0.2
1,25(OH)_2_D, pmol/L	0.014 (0.011)	0.2	0.012 (0.021)	0.6	0.045 (0.035)	0.2	−0.005 (0.036)	0.9
PTH, ng/L	−3.365 (2.745)	0.2	4.628 (2.601)	0.08	8.371 (4.443)	0.06	−2.097 (4.266)	0.6
DBP, mg/L	0.018 (0.011)	0.1	0.027 (0.023)	0.2	−0.007 (0.030)	0.8	0.040 (0.028)	0.2

^1^Linear mixed model to determine predictors of 25(OH)D_3_ half-life in each group. Fixed effects were group (pregnancy/lactation or NPNL) and time (Phase 1 and 2). Participant ID was included as a random effect. Reported for each group are the β coefficient (slope), standard error (SE) of β and associated *P* value.
